# Rhinovirus species and tonsillar immune responses

**DOI:** 10.1186/s13601-019-0302-7

**Published:** 2019-12-02

**Authors:** Emilia Mikola, Oscar Palomares, Riitta Turunen, Matti Waris, Lotta E. Ivaska, Antti Silvoniemi, Tuomo Puhakka, Beate Rückert, Tytti Vuorinen, Mübeccel Akdis, Cezmi A. Akdis, Tuomas Jartti

**Affiliations:** 1 Department of Otorhinolaryngology, Turku University Hospital and University of Turku, Turku, Finland; 2 Swiss Institute of Allergy and Asthma Research, University of Zürich, Davos, Switzerland; 3 Christine Kühne-Center for Allergy Research and Education, Davos, Switzerland; 4 Department of Biochemistry and Molecular Biology, School of Chemistry, Complutense University of Madrid, Madrid, Spain; 5 Department of Pediatrics and Adolescent Medicine, Turku University Hospital and University of Turku, Turku, Finland; 6 Clinical Microbiology, Turku University Hospital, Turku, Finland; 7 Institute of Biomedicine, University of Turku, Turku, Finland; 8 Department of Otorhinolaryngology, Satakunta Central Hospital, Sairaalantie 3, 28500 Pori, Finland; 9 Children´s Hospital, Helsinki University Hospital and University of Helsinki, Helsinki, Finland

**Keywords:** Allergy, Asthma, Children, Cytokine, Rhinovirus, Tonsil

## Abstract

**Background:**

Rhinovirus A and C infections are important contributors to asthma induction and exacerbations. No data exist on the interaction of local immune responses in rhinovirus infection. Therefore, we aimed to determine the tonsillar immune responses according to rhinovirus A, B and C infections.

**Methods:**

We collected tonsillar samples, nasopharyngeal aspirates and peripheral blood from 42 rhinovirus positive tonsillectomy patients. Fifteen respiratory viruses or their types were investigated from nasopharynx and tonsil tissue, and rhinovirus species were typed. The expression of 10 cytokines and 4 transcription factors (IFN-α, IFN-β, IFN-γ, IL-10, IL-13, IL-17, IL-28, IL-29, IL-37, TGF-β, FOXP3, GATA3, RORC2 and Tbet) were studied from tonsil tissue by quantitative PCR. A standard questionnaire of respiratory symptoms and health was filled by the patient or his/her guardian. The patients were divided into three groups by the determination of rhinovirus species.

**Results:**

Overall, 16 patients had rhinovirus A, 12 rhinovirus B and 14 rhinovirus C infection. In rhinovirus B positive group there were significantly less men (*P* = 0.0072), less operated in spring (*P* = 0.0096) and more operated in fall (*P* = 0.030) than in rhinovirus A or C groups. Rhinovirus A positive patients had more respiratory symptoms (*P* = 0.0074) and particularly rhinitis (*P* = 0.036) on the operation day. There were no significant differences between the groups in virus codetection. In adjusted analysis, rhinovirus C infections were associated with increased IFN-α (*P* = 0.045) and decreased RORC2 expression (*P* = 0.025).

**Conclusions:**

Rhinovirus species associated differently with clinical characteristics and tonsillar cytokine responses.

## Background

Human rhinovirus (RV) is a positive-strand RNA-virus in the family *Picornaviridae* and genus Enterovirus [1, 2]. Three species have been found, A and B in the 1950s and C in 2006 after the development of highly sensitive molecular techniques [1, 3]. RV is widely known to be a major cause of common cold and upper respiratory illnesses [1, 4–6] but it is also proven to cause lower respiratory diseases [1, 4–6]. There is evidence that it is a contributor to asthma induction and exacerbations [1, 4–6]. Especially RV-A and RV-C seem to cause more severe respiratory illnesses and are dominate over RV-B in patients with asthma or chronic obstructive pulmonary disease [1, 2, 4, 7]. Moreover RV-A and RV-C are common in hospitalized children [5, 7, 8].

RV infection causes cell destruction and changes in immunological reactions [6, 8]. It has influenced the expression of several interferons and cytokines such as IL (interleukin) -4, IL-6, IL-8, IL-13, IL-16 and IFN (interferon)-γ [1, 6, 8]. Differences between the immune responses of the three species has not been extensively studied. Jong et al. [9] found no significant differences in the cytokine levels of nasopharyngeal aspirate of RV-infected children but they studied only four cytokines (IFN-γ, IL-4, IL-10 and tumor necrosis factor α). It seems that RV-B replicates more slowly and induces less cytokine production than RV-A and RV-C [10].

In our previous studies [11, 12] we have regarded tonsils as a good in vivo—model for investigating immune responses. Tonsils are local lymphoid tissue and in close contact with infective agents and allergens. In this study our aim was to observe the differences in tonsillar cytokine expression between RV-A, -B and -C species in routine tonsillectomy patients. Most previous studies concern only cytokine expression of nasopharyngeal aspirate in hospitalized patients.

## Methods

### Patients

We enrolled 200 patients who were going through elective tonsillectomy or adenotomy between April 2008 and March 2009 due to clinical indication for the operation in Satakunta Central Hospital, Pori Finland. Written consent to participate in the study from the patient or his/her guardian was considered as inclusion criteria along with the tonsillectomy. The study protocol was approved by the Ethics Committee of Satakunta Central Hospital.

### Study protocol

From the enrolled patients we collected samples from tonsils, nasopharyngeal aspirate and peripheral blood. The tonsillectomy was performed according to clinical routine. Tonsil samples were cut into 3–4 mm pieces and stored in RNA*later* RNA stabilization reagent (Qiagen, Hilden, Germany), incubated + 4 °C until next working day and after removal of non-absorbed reagent stored in − 80 °C. Nasopharyngeal aspirates were collected at the beginning of the operation during anesthesia using a standardized procedure [11]. For the viral analysis, part of the tonsil and nasopharyngeal aspirate (NPS) were stored in dry tubes at − 80 °C [11]. Serum 25(OH)D measurement and serum IgE measurements for food allergen and aeroallergen screening (Phadiatop Combi^®^, Phadia, Uppsala, Sweden) were made from peripheral blood samples. Patient or his/her guardian also filled a standard questionnaire concerning their respiratory symptoms 30 days prior the operation and allergic diseases (Additional file 1: Table S1).

### Analysis of viruses and cytokines

In-house real-time PCR was used to detect enterovirus (EV), human bocavirus-1 (HBoV-1), respiratory syncytial virus (RSV) and RV [11]. For detection of adenovirus (AdV), coronavirus (CoV), influenza A and B viruses (Flu A and B), metapneumovirus (MPV), parainfluenza virus types 1–3 (PIV 1–3), RSV group A and B, and RV Seeplex RV12 ACE detection multiplex PCR assay was used (Seegene, Seoul, Korea) [11, 13]. Virus diagnostics were performed in the Department of Virology, University of Turku, Turku, Finland, and in the Department of Clinical Microbiology, Karolinska University Hospital, Stockholm, Sweden. Rhinovirus typing was made by amplifying and sequencing the partial VP4/VP2 and 5′ non coding region of RV genome [14, 15].

Previously stabilized tonsil tissue were homogenized in grinding tubes containing CK28 ceramic beads by using a Precellys 24 homogenizer (Bertin Technologies, Montigny le Bretonneux, France) two times at 6000 rpm for 50 s [11]. Total RNA was then isolated using the RNeasy mini kit (Qiagen, Hilden, Germany). Reverse transcription was performed with the Revert Aid M-MuLV Reverse Transcriptase (Fermentas, St. Leon-Rot, Germany) using random hexamer primers according to the manufacturers protocol. Gene expression of IFN-α, IFN-β, IFN-γ, IL-10, IL-13, IL-17, IL-28, IL-29, IL-37, TGF-β (tumour growth factor β), FOXP3 (forkhead box protein 3), GATA3 (GATA-binding factor 3), RORC2 (RAR-related orphan receptor C 2) and Tbet (T-box transcription factor) were analyzed by quantitative real-time PCR using iTaq SYBR Green Supermix with ROX (Bio-Rad, Hercules, CA, USA) on a 7900HT Fast Real-Time PCR instrument (Applied Biosystems, Foster City, CA, USA). Housekeeping elongation factor 1α (EF1α) was used for normalization. Data are shown as relative expressions, which show 2^−(ΔCT)^ values multiplied by 10^4^, where ΔCT corresponds to the difference between the CT value for the gene of interest and EF1α [11, 12].

### Statistical analysis

Data was analyzed using JMP Pro version 12.0.1 software (SAS Institute Inc. Cary, NC, USA). Due to skewed distribution continuous variables are described as medians and interquartile ranges and were analyzed using Kruskal–Wallis test. Categorical variables are expressed as frequencies and percentages and were analyzed using Chi square test or Fisher´s exact test (when counts < 5). Before regression analyses, cytokine and transcription factor values were log-transformed because of positively skewed distributions. Clinical, viral and immunological differences between study groups were analyzed using unadjusted and multivariable linear model analysis. The adjustments for immunologic analyses included clinical factors and virus infections which significantly differed between the groups (sex, season of the surgery spring or fall, any respiratory symptoms on the operation day, rhinitis on the operation day) and age. Backward stepwise method was used for the final adjustment model separately for each cytokine and transcription factor. Only statistically significant factors were kept in the model. *P* < 0.05 was considered statistically significant.

## Results

### Recruitment and study population

**Fig. 1:**
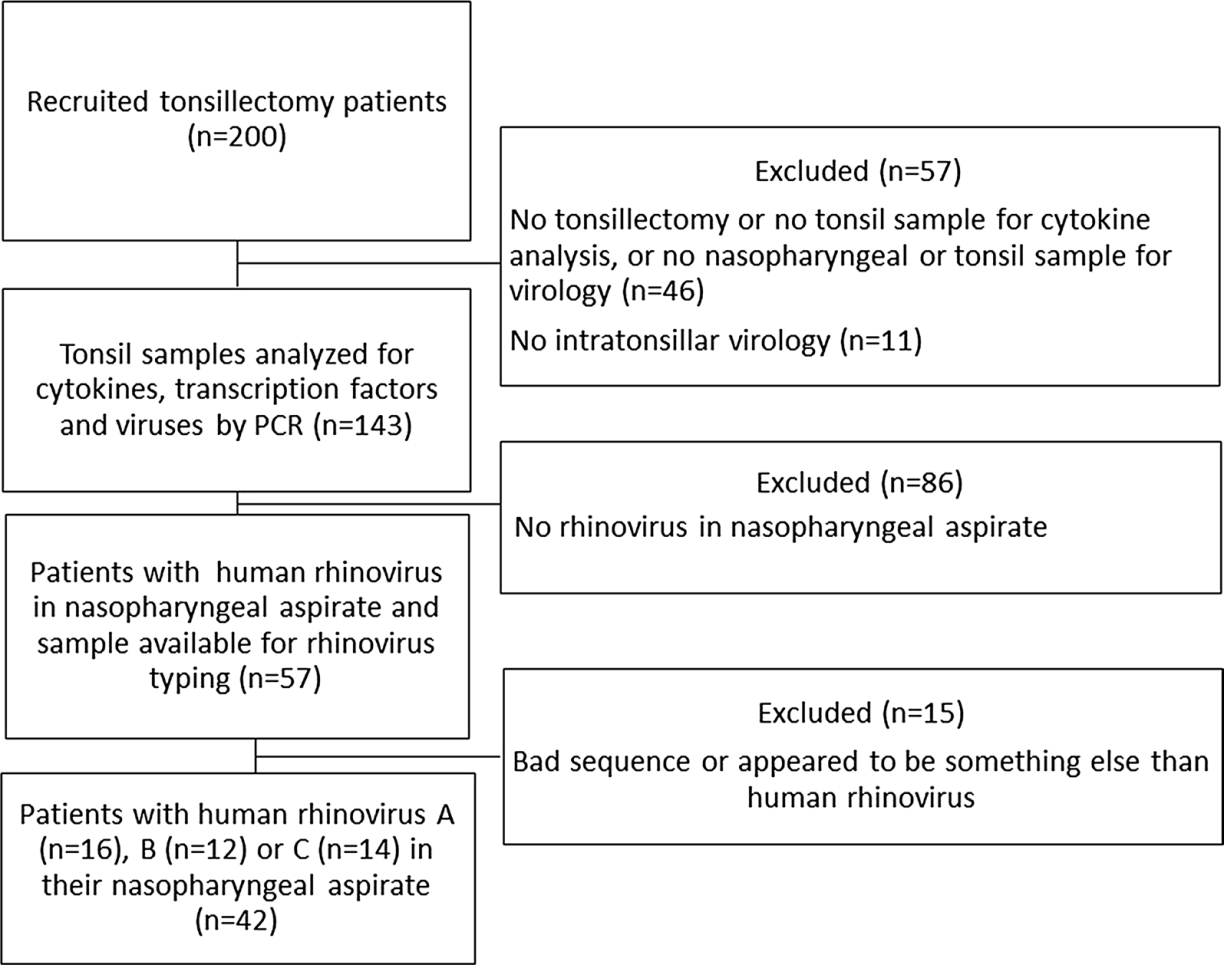
Study flow chart

Of 200 patients 143 had viral and immunological analyzes made from their tonsils. Fifty-seven patients were excluded because of poor quality of tonsil samples (Fig. 1). Of those 143 patients, fifty-seven samples were rhinovirus positive in primary PCR test. Of them rhinovirus sequencing was successful from 42 samples (Fig. 1).

### Patient characteristics

Median age of study subjects was 9.3 years, 55% were males, and 42% had food or aeroallergen sensitization (Table 1). Main indications for tonsillectomy were tonsillar hypertrophy in 43% subjects and recurrently infected tonsils in 19% subjects (Table 1). RV-A and -C groups had male dominance. RV-A epidemic occurred in spring and RV-C epidemics in fall, and RV-A infected subjects were most often symptomatic (Table 1).

**Table 1:** Patient characteristics

Characteristics	All, n = 42	RV-A, n = 16	RV-B, n = 12	RV-C, n = 14	*P* value
Age, years	9.3 (2.6, 40)	9.1 (2.6, 17)	13.3 (4.8, 36)	8.5 (3.2, 40)	0.38
Male	23 (55%)	11 (69%)	2 (17%)	10 (71%)	*0.0072*
Tonsillectomy and adenotomy	22 (52%)	9 (56%)	6 (50%)	7 (50%)	0.93
Self-smoking	1/41 (2%)	0 (0%)	1 (8.3%)	0/13 (0%)	0.29
Maternal smoking	16/41 (39%)	6 (38%)	4/11 (36%)	6 (43%)	1.0
Paternal smoking	17/37 (46%)	5/15 (33%)	5/10 (50%)	7/12 (58%)	0.42
Season of the surgery					
Winter (months 12–2)	4 (9.5%)	0 (0%)	3 (25%)	1 (7.1%)	0.057
Spring (months 3–5)	11 (26%)	8 (50%)	0 (0%)	3 (21%)	*0.0096*
Summer (months 6–8)	6 (14%)	4 (25%)	0 (0%)	2 (14%)	0.21
Fall (months 9–11)	21 (50%)	4 (25%)	9 (75%)	8 (57%)	*0.030*
Indication of the surgery					
Obstruction only	18 (43%)	5 (31%)	6 (50%)	7 (50%)	0.49
Recurrent tonsillitis only	8 (19%)	2 (13%)	2 (17%)	4 (29%)	0.55
Obstruction + tonsillitis	7 (17%)	3 (19%)	2 (17%)	2 (14%)	1.0
Other	9 (21%)	6 (38%)	2 (17%)	1 (7.1%)	0.14
Respiratory symptoms on operation day	13/39 (33%)	10 (63%)	1/10 (10%)	2/13 (15%)	*0.0074*
Throat pain	3/39 (7.7%)	2 (13%)	0/10	1/13 (7.7%)	0.77
Cough	6/39 (15%)	4 (25%)	1/10 (10%)	1/13 (7.7%)	0.53
Acute otitis media	0/39 (0%)	0 (0%)	0/10 (0%)	0/13 (0%)	–
Wheezing	0/39 (0%)	0 (0%)	0/10 (0%)	0/13 (0%)	–
Other	1/39 (2.6%)	1 (6.3%)	0/10 (0%)	0/13 (0%)	1.0
Respiratory symptoms within 2 weeks	18/34 (53%)	10/15 (67%)	4/8 (50%)	4/11 (36%)	0.34
Respiratory symptoms within 4 weeks	22/34 (65%)	11/15 (73%)	5/8 (63%)	6/11 (55%)	0.66
Total 25-OHD (nmol/l)	54 (45, 66)	54 (48, 68)	51 (38, 64)	58 (41, 69)	0.72
Free	7.2 (5.3, 8.2)	7.0 (5.7, 10)	6.5 (4.3, 7.9)	7.3 (5.3, 8.5)	0.69
Bioavailable	2.4 (1.9, 3.0)	2.5 (2.0, 4.0)	2.1 (1.6, 2.7)	2.4 (1.8, 3.3)	0.45
Self-reported allergy	18 (43%)	6 (38%)	7 (58%)	5 (36%)	0.44
Physician-diagnosed atopic dermatitis	7/41 (17%)	1 (6.3%)	4 (33%)	2/13 (15%)	0.19
Self-reported allergic rhinitis	10/40 (25%)	4 (25%)	4/11 (36%)	2/13 (15%)	0.53
Physician-diagnosed asthma	8/40 (20%)	1/15 (6.7%)	5 (42%)	2/13 (15%)	0.099
Sensitization	14/33 (42%)	3/11 (27%)	5/9 (56%)	6/13 (46%)	0.45
Food	4/33 (12%)	1/11 (9.1%)	0/9 (0%)	3/13 (23%)	0.42
Aeroallergen	11/33 (33%)	2/11 (18%)	5/9 (56%)	4/13 (31%)	0.25

25-OHD, 25-hydroxyvitamin D

Data are expressed as median (range), or number of subjects (%)

### Viral findings

Sixteen (38%) patients had positive RV-A, 12 (29%) positive RV-B and 14 (33%) positive RV-C detection in NPS (Table 2). Most common virus codetections were HBoV-1 (19%), AdV (7%), CoV (7%) in NPS and HBoV-1(17%), AdV (10%), EV (10%), PIV 1-3 (7%) in tonsils (Table 2). Other codetections in NPS were EV, Flu A or B, PIV 1–3 and RSV and in tonsils CoV, MPV and RSV (all < 5%). In tonsils RV was found from one patient in each group. There were no statistically significant differences in virus codetection between the RV species (Table 2).

**Table 2:** Virus detection

Virus	Nasopharynx			*P* value	Tonsil			*P* value
	RV-A n = 16	RV-B n = 12	RV-C n = 14		RV-A n = 16	RV-B n = 12	RV-C n = 14	
Adenovirus	1 (6.3%)	2 (17%)	0 (0%)	0.27	3 (19%)	0 (0%)	1 (7.1%)	0.36
Bocavirus-1	2 (13%)	3 (25%)	3 (21%)	0.70	5 (31%)	1 (8.3%)	1 (7.1%)	0.22
Coronavirus	3 (19%)	0 (0%)	0 (0%)	0.10	1 (6.3%)	0 (0%)	0 (0%)	1.0
Enterovirus	1 (6.3%)	0 (0%)	0 (0%)	1.0	1 (6.3%)	1 (8.3%)	2 (14%)	0.82
Influenza A or B virus	1 (6.3%)	0 (0%)	0 (0%)	1.0	0 (0%)	0 (0%)	0 (0%)	–
Metapneumovirus	0 (0%)	0 (0%)	0 (0%)	–	1 (6.3%)	0 (0%)	0 (0%)	1.0
Parainfluenza virus types 1–3	2 (13%)	0 (0%)	0 (0%)	0.32	2 (13%)	0 (0%)	1 (7.1%)	0.77
Respiratory syncytial virus	1 (6.3%)	0 (0%)	0 (0%)	1.0	1 (6.3%)	0 (0%)	0 (0%)	1.0
Rhinovirus	16 (100%)	12 (100%)	14 (100%)	–	1 (6.3%)	1 (8.3%)	1 (7.1%)	1.0
Number of positive viruses								
1 virus	8 (50%)	8 (67%)	11 (79%)	0.26	4 (25%)	1 (8.3%)	6 (43%)	0.15
2 viruses	5 (31%)	3 (25%)	3 (21%)	0.91	2 (13%)	1 (8.3%)	0 (0%)	0.49
3 viruses	3 (19%)	1 (8.3%)	0 (0%)	0.29	1 (6.3%)	0 (0%)	0 (0%)	1.0
4 viruses	0 (0%)	0 (0%)	0 (0%)	–	1 (6.3%)	0 (0%)	0 (0%)	1.0
≥ 1 viruses	16 (100%)	12 (100%)	14 (100%)	–	8 (50%)	2 (17%)	6 (43%)	0.18
≥ 2 viruses	8 (50%)	4 (33%)	3 (21%)	0.26	4 (25%)	1 (8.3%)	0 (0%)	0.11
≥ 3 viruses	3 (19%)	1 (8.3%)	0 (0%)	0.29	2 (13%)	0 (0%)	0 (0%)	0.32
≥ 4 viruses	0 (0%)	0 (0%)	0 (0%)	–	1 (6.3%)	0 (0%)	0 (0%)	1.0

Data are expressed as number of subjects (%)

### Cytokines and transcription factors

**Fig. 2:**
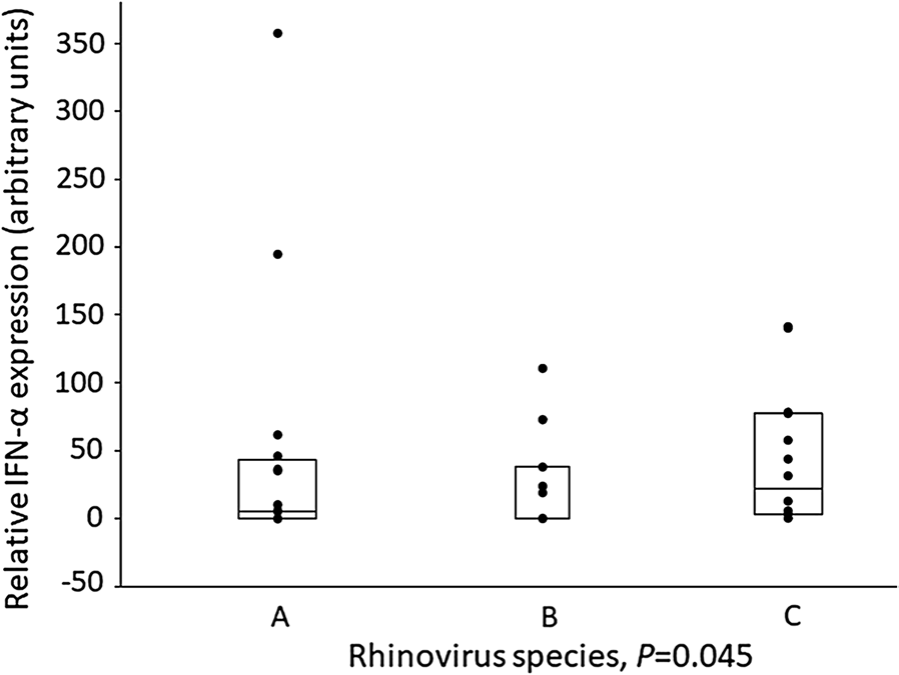
Relative tonsillar expression of IFN-α. Comparison of tonsil samples between 16 RV-A, 12 RV-B and 14 RV-C positive patients (Table 3). Values are arbitrary units × 10^4^ relative to EF1α. Data represents median with interquartile range. The median of RV-B is 0.37. IFN, interferon

**Fig. 3:**
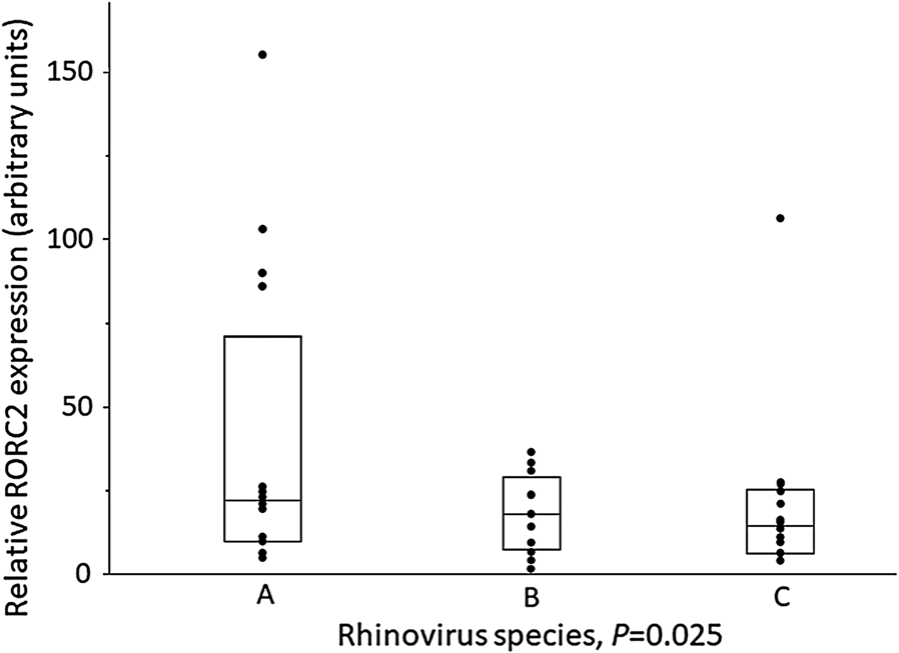
Relative tonsillar expression of RORC2. Comparison of tonsil samples between 16 RV-A, 12 RV-B and 14 RV-C positive patients (Table 3). Values are arbitrary units × 10^4^ relative to EF1α. Data represents median with interquartile range. RORC2, RAR-related orphan receptor C 2

**Fig. 4:**
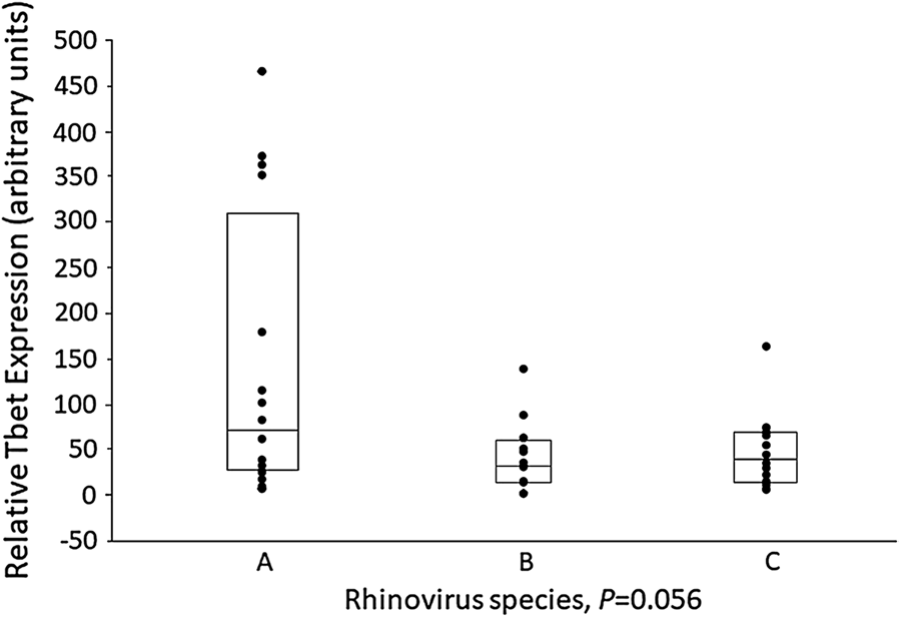
Relative tonsillar expression of Tbet. Comparison of tonsil samples between 16 RV-A, 12 RV-B and 14 RV-C positive patients (Table 3). Values are arbitrary units × 10^4^ relative to EF1α. Data represents median with interquartile range. Tbet, T-box transcription factor

Table 3 presents the expression rates of cytokines and transcription factors, including T-helper_1_, T-helper_2_, T-helper_17_ and T-regulatory type cytokines and transcription factors and type I/III interferons. In adjusted analysis, the highest IFN-α expression and the lowest RORC2 expression were associated with RV-C detection (both *P* < 0.05 for overall differences) (Figs. 2 and 3). RV-B detection was associated with lowest Tbet expression (*P* = 0.056 for overall differences) (Fig. 4). Otherwise, no significant differences or tendencies were found (Table 3).

**Table 3:** Cytokine or transcription factor expression

Cytokine or transcription factor	RV-A n = 16	RV-B n = 12	RV-C n = 14	*P* value univariate	*P* value multivariate	Adjustments
Th_1_ -type						
IFN-γ	71* (27, 104)	58 (34, 72)	72 (33, 98)	0.99	0.61	Spring
Tbet	72 (27, 309)	33 (15, 60)	40 (15, 70)	0.082	0.056	Respiratory symptoms, spring
Th_2_ -type						
IL-13	0.40 (0.018, 5.0)	0.37 (0.020, 3.4)	1.8 (0.36, 7.6)	0.14	0.14	–
GATA3	31 (13, 50)	20 (6.2, 33)	21 (12, 37)	0.36	0.98	Spring
Th_17_ -type						
IL-17	15 (9.1, 26)	14 (3.8, 26)	8.0 (4.0, 14)	0.37	0.36	Age
RORC2	22 (9.7, 71)	18 (7.2, 29)	14 (6.2, 25)	0.31	*0.025*	Respiratory symptoms
Treg -type						
IL-10	55 (32, 84)	40 (14, 62)	33 (22, 74)	0.34	0.96	Age, spring
IL-37	0.38 (0.14, 0.50)	0.22** (0.14, 0.34)	0.27 (0.14, 0.34)	0.64	0.11	Respiratory symptoms
FOXP3	56 (19, 107)	33 (13, 89)	29 (16, 88)	0.47	0.12	Rhinitis
TGF-β	146 (94, 185)	192 (108, 252)	139 (102, 187)	0.49	0.49	–
Type I/III interferons						
IFN-α	5.8 (0, 44)	0.37** (0.33, 38)	22 (2.9, 78)	0.23	*0.045*	Respiratory symptoms
IFN-β	15 (3.3, 81)	4.9 (2.2, 68)	61 (7.7, 116)	0.30	0.30	–
IL-28	25* (1.4, 88)	9.7 (1.4, 62)	32 (13, 109)	0.41	0.41	–
IL-29	5.9 (1.3, 35)	3.1 (1.3, 22)	11 (4.4, 40)	0.53	0.53	–

*IFN* interferon, *Tbet* T-box transcription factor, *IL* interleukin, *GATA3* GATA-binding factor 3, *RORC* RAR-related orphan receptor C, *FOXP* forkhead box protein, *TGF* tumour growth factor, *Th* T helper cell, *Treg* T regulatory cell

Values are arbitrary units × 10^4^ relative to EF1α

Data are expressed as median (interquartile range)

Adjustments are selected backward stepwise from significant differences between groups (sex, rhinitis on the operation day, any respiratory symptoms on the operation day, operation made spring, operation made fall) and age

*n = 15

**n = 11

## Discussion

The clinical consequences of rhinovirus A, B and C infections have previously been proven to differ from each other [1]. Thus, it is evident that immunological responses due to these distinct species are diverse. Bearing these things in mind, we conducted our study with routine tonsillectomy patients, which has three main findings. First, rhinovirus species A, B and C were rather equally distributed among relatively asymptomatic tonsillectomy patients and were mainly found in nasopharyngeal aspirate compared to the low persistence rate in tonsils. Second, rhinovirus species were differently associated with tonsillar cytokine responses. Rhinovirus C affected patients had increased IFN-α and decreased RORC2 expression in tonsils when compared to other rhinovirus species (Figs. 2 and 3). Third, rhinovirus species were differently associated with clinical characteristics.

The similar incidence rates of RV species was a bit unexpected since RV-B has usually been slightly less prevalent than RV-A and RV-C in healthy subjects [5, 7, 8]. However, this difference has been more pronounced in children with severe wheezing and asthma, in whom RV-A and RV-C species have also associated with more severe symptoms [2, 7, 8]. Our children study subjects were rather young (median 9 years old) and generally healthy which is likely to favor more equal distribution of RV species. Symptomatic and asymptomatic infections are also more common in children than in adults [1, 11].

We found no difference between rhinovirus A, B or C positive tonsillectomy patients in terms of viral coinfections. It has been shown that viral codetection is common especially in children [16, 17]. Morikawa et al. [16] noted that the coinfection rates of RV-A and C are high, but it remains unclear whether they found differences between the coinfection rates of RV-A, RV-B and RV-C. Miller et al. [18] found more coinfections with RV-A than with RV-C in hospitalized children. We found male sex to be dominant in RV-A and RV-C infected patients. This is in line with a previous study with male sex dominating in RV-A and RV-C infections in hospitalized children [14]. Reason for that might be in sex hormones as androgens promote Th1 (T helper type 1 cell) responses and estrogen and progesterone Th2 responses [19]. The seasonality of all the rhinovirus types seemed to follow the seasonality described in literature [2, 8, 16, 20, 21]: there was a major peak in fall and a smaller peak in spring, but the rhinoviruses were detected throughout the whole year. RV-C and RV-B were found mostly from patients operated in fall whereas RV-A was most commonly found in spring.

The most interesting and novel finding was the higher level of tonsillar IFN-α production in patients with RV-A and RV-C infection, especially in those with RV-C, compared with RV-B infected patients. Type I interferons, including IFN-α, are important antiviral cytokines and for preventing inappropriate Th2 response [8, 22]. Considering the ability of RV-A and RV-C to cause more severe infection than RV-B [1, 2], it is plausible that they may also induce stronger interferon responses. In fact, strong IFN-γ responses have been associated with reduced virus shedding, and exposure to IFN-α or IFN-γ has limited RV infection in vitro [6, 21]. Our findings are in agreement with these previous findings and extend the increased expression of type I interferon to local lymphoid tissue level in healthy individuals. Moreover, some previous findings suggest that RV-C may cause slightly more severe infection than RV-A [1] which also fit to our finding.

In addition, we found a close to significant difference between the Tbet expressions in tonsils of the patients harboring distinct RV species (Fig. 4). The level of expression was highest in patients with RV-A infection and lowest in those with RV-B infection. Tbet controls the differentation of Th1 cells and acts together with RUNX3 (runt-related transcription factor) to induce IFN-γ production [23]. Glanville et al. [24] found Tbet deficient mice to develop Th2 and Th17 responses to RV infection instead of Th1 responses. They also found Tbet deficient mice to develop significant eosinophilia and mucus production after RV infection. Our finding on Tbet is in agreement with previous experimental and clinical data. The higher Tbet expression supports also the decline of RORC2 in RV-C group being also in agreement with the findings of Glanville et al. [24]. RORC2 acts as a transcription factor for IL-17 which is involved in many inflammatory disorders [23].

The rate of respiratory symptoms on the operation day was fairly small (33%) thus our patients are representing mostly asymptomatic subjects. More than half had symptoms 2 to 4 weeks prior the operation which might reflect virus shedding from a past symptomatic infection. It may take even 5–6 weeks from rhinovirus to disappear from nasal mucus [25]. Even in healthy children rhinovirus have been found in 15 to 35% in asymptomatic individuals [2, 20, 21, 26, 27] giving an explanation of why only a small proportion of rhinovirus infected patients had symptoms on the operation day. Majority of the patients having respiratory symptoms on the operation day had RV-A or RV-C infection and only one had RV-B infection supporting the stronger clinical importance of RV-A and RV-C.

The strength of the study was that it consisted relatively healthy tonsillectomy patients, and thereby our results suggest normal cytokine responses due to symptomatic or asymptomatics RV infection. Many previous studies concern hospitalized patients having an acute infection [7, 9, 14, 21, 28]. Also, our study population was constructed with both children and adults whereas often studies concern only children. We also used single cytokine PCR and patients as well as their preceding symptoms were well-characterized. There were also some limitations. Sample size was relatively small and because of subpopulation of study subjects the study was not powered to detect significant changes in outcome measures. We studied only viruses, not bacteria. As there was no control group the observations are not clearly associated with the presence of an acute infection. Our results can only be generalized for healthy individuals. Then, the immune response due to RV-infection is a dynamic process and the relevance of history of previous RV infections remains obscure. The samples were taken at a single time point as the tonsils can be removed only once and therefore it is difficult to assess whether the observed changes were time-dependent or uniform over longer period of time.

## Conclusions

To our best knowledge, this was the first time to study immune responses between rhinovirus species at the local lymphoid tissue level. We found that rhinovirus species were associated differently with clinical characteristics and tonsillar cytokine responses. The most interesting finding was the increase of IFN-α and Tbet and decrease of RORC2 in rhinovirus C affected patients suggesting that rhinovirus C infection has greater effect on tonsillar Th1 and Th17 responses than rhinovirus A or B infection. Our results encourage to study immune responses at the local lymphoid tissue level by using tonsils.

## Supplementary information

**Additional file 1: Table S1.** Health questionnaire.

## Data Availability

The datasets generated and analysed during the current study are not publicly available due to individual privacy but are available from the corresponding author on reasonable request.

## Abbreviations

AdV: adenovirus
CoV: coronavirus
EF1α: housekeeping elongation factor 1α
EV: enterovirus
Flu: influenza virus
FOXP: forkhead box protein
GATA: GATA-binding factor
HBoV: human bocavirus
IFN: interferon
IgE: immunoglobulin E
IL: interleukin
IQR: interquartile range
MPV: metapneumovirus
NPS: nasopharyngeal aspirate
PCR: polymerase chain reaction
PIV: parainfluenza virus
RORC: RAR-related orphan receptor C
RNA: ribonucleic acid
RSV: respiratory syncytial virus
RUNX: runt-related transcription factor
RV: human rhinovirus
Tbet: T-box transcription factor
TGF: tumour growth factor
Th: T helper cell
